# Evaluation of a community-based ART programme after tapering home visits in rural Sierra Leone: a 24-month retrospective study

**DOI:** 10.1080/17290376.2018.1527244

**Published:** 2018-09-26

**Authors:** J. Daniel Kelly, Raphael Frankfurter, Gregoire Lurton, Sulaiman Conteh, Susannah F. Empson, Fodei Daboh, Brima Kargbo, Thomas Giordano, Joia Mukherjee, M. Bailor Barrie

**Affiliations:** a School of Medicine, University of California at San Francisco, San Francisco, CA, USA; b National HIV/AIDS Secretariat, Freetown, Sierra Leone; c Wellbody Alliance, Koidu Town, Sierra Leone; d Department of Global Health, University of Washington, Seattle, WA, USA; e Department of Medicine, Baylor College of Medicine, Houston, TX, USA; f Division of Global Health Equity, Brigham and Women’s Hospital, Boston, MA, USA; g Department of Global Health and Social Medicine, Harvard Medical School, Boston, MA, USA

**Keywords:** HIV/AIDS, community-based ART programme, adherence, retention in care, Sierra Leone

## Abstract

Evaluations of community-based antiretroviral therapy (ART) programmes have demonstrated positive outcomes, but little is known about the impact of tapering community-based ART. The objective of this study was to assess 24-month HIV retention outcomes of a community-based ART programme and its tapered visit frequency in Koidu City, Sierra Leone. This retrospective, quasi-experimental study compared outcomes of 52 HIV-infected persons initiated on community-based ART against 91 HIV-infected persons receiving the standard of care from November 2009 to February 2013. The community-based ART pilot programme was designed to strengthen the standard of care through a comprehensive, patient-centred case management strategy. The strategy included medical, educational, psychological, social, and economic support. Starting in October 2011, the frequency of home visits was tapered from twice daily every day per week to once daily three days per week. Outcomes were retention in care at 12 and 24 months and adherence to ART over a three-month time period. Participants who received community-based ART had significantly higher retention than those receiving standard of care. At 12 months, retention rates for community-based ART and standard of care were 61.5% and 31.9%, respectively (*p* < .01). At 24 months, retention rates for community-based ART and standard of care were 73.1% and 44.0%, respectively (*p* < .01). Significant differences in levels of adherence were observed when comparing community-based ART against persons receiving standard of care (*p* < .05). No differences in adherence levels were observed between groups of people receiving various frequencies of home visits. Our pilot programme in Koidu City provides new evidence that community-based ART has the potential to improve retention and adherence outcomes for HIV-infected persons, regardless of the frequency of home visits. Overcoming the barriers to HIV care requires a comprehensive, patient-centred approach that may include clinic-based and community-based interventions.

## Introduction

Since the early 2000s, antiretroviral therapy (ART) has been made available throughout the world. As UNAIDS promotes a strategy to have 90% of HIV-infected persons virally suppressed (UNAIDS, 2014), structural barriers to care impede progress towards effective and universal HIV care (Gill, Hamer, Simon, Thea, & Sabin, 2005; Piot et al., 2015). In settings of sub-Saharan Africa such as Sierra Leone (Slaymaker et al., 2017), where the effective HIV care is poor (23%) (Kelly et al., 2016), it is crucial to refine retention and adherence interventions in order to end the HIV epidemic (Geng et al., 2013; Ware et al., 2013).

While some clinic-based strategies such as early initiation of ART have improved retention in care (Brown et al., 2016), other clinic-based strategies such as peer support or innovative counselling programmes have lacked anti-stigma or health outcome benefits, respectively (Rao et al., 2018; Uusküla et al., 2018). Some HIV-infected persons who take ART face barriers to care and require accompaniment in the community for various reasons, ranging from a weak health system, stigma, social ostracisation, alcohol abuse, as well as competing demands on time and money in the context of widespread abject poverty (Fatti, Meintjes, Shea, Eley, & Grimwood, 2012; Munyaneza et al., 2018; Pokhrel, Gaulee Pokhrel, Neupane, & Sharma, 2018; Vogt et al., 2017). Younger age groups are particularly in need of community-based ART (Fatti et al., 2018; Grimwood et al., 2012). Studies have found that community-based ART programmes have addressed these social barriers to care, promoted retention in care and adherence to ART, and have thus demonstrated positive clinical outcomes (Mukherjee, Ivers, Leandre, Farmer, & Behforouz, 2006; Rich et al., 2012). In order to reach our goal of 90-90-90, it will be essential to employ a combination of clinic-based and community-based strategies that facilitates universal access to care (Piot et al., 2015; UNAIDS, 2014, 2015a).

There has been a shift from directly observed therapy (DOT) to community-based ART as a key component of national HIV programmes (Hart et al., 2010; Rich et al., 2012). A meta-analysis of DOT-ART substantiated impacts of community-based ART on virologic, immunologic, and adherence outcomes (Hart et al., 2010). By including studies beyond just randomised controlled trials, the review attempted to describe ‘real-world’ conditions, yet few studies have described community-based ART programmes for more than 12 months (Rich et al., 2012). Lessons from community-based ART have suggested that some individuals may need long-term DOT-ART as part of a comprehensive approach to HIV care (Farmer et al., 2001; Koenig et al., 2006). Sustaining DOT-ART for prolonged periods, however, may be a barrier to routine implementation of such approaches, and one possible solution to that challenge is to take a patient-centred, individually attentive approach to implementing DOT-ART through community-based ART programmes (Shanaube & Bock, 2015). There is evidence that longer-term community-based ART programmes may be associated with positive health outcomes (Fatti et al., 2012), but evidence about community-based ART and the impact of tapering home visits on health outcomes is lacking.

In collaboration with the National HIV/AIDS Secretariat of Sierra Leone, the Kono District Health Management Team, and Wellbody Alliance, we initiated a pilot programme in Koidu City, Sierra Leone, in November 2009 that enhanced the standard of HIV care through accompaniment, transportation of acutely ill patients, and comprehensive medical support. In October 2011, we reduced home visits of our community-based ART programme to three times per week due to multiple factors, including CHW burnout, CHW-client logistical constraints, supervisory challenges, growth of the patient pool without parallel growth in programme, and funding constraints. We chose to transition the programme to three home visits per week based on the success of another community-based ART programme in Liberia (Lee et al., 2011). Since then, the National HIV/AIDS Secretariat has decided to use our programme as a model for national scale-up. The objective of this study was to assess 24-month HIV retention outcomes of a community-based ART programme and its tapered visit frequency in Koidu City, Sierra Leone.

## Methods

### Study design, setting and study population

We used a retrospective, quasi-experimental study design to compare outcomes of HIV-infected persons who received community-based ART with persons only receiving the standard of care in rural Kono District, Sierra Leone. Starting in November 2009, we collected time-matched data for participants who received standard of care with those who also received community-based ART. We enrolled participants in our study until February 2011. We censored participants at 24 months, ending data collection in March 2013. See Table 1.

**Table 1.** Study timeline.

Years	Year 1 (11/09–10/10)				Year 2 (11/10–10/11)				Year 3 (11/11–10/12)				Year 4 (11/12–03/13)	
Quarters	1	2	3	4	1	2	3	4	1	2	3	4	1	2
Enrollment	x	x	x	x	x	x								
Standard of care	x	x	x	x	x	x	x	x	x	x	x	x	x	x
Retention data	x	x	x	x	x	x	x	x	x	x	x	x	x	x
Adherence data				x	x	x	x	x	x	x				
Home visits 2×/day 7×/week	x	x	x	x	x	x	x	x						
Retention data	x	x	x	x	x	x	x	x						
Adherence data				x	x	x	x	x						
Home visits 1×/day 3×/week								x	x	x	x	x	x	x
Retention data								x	x	x	x	x	x	x
Adherence data								x	x	x				

Sierra Leone is one of the poorest countries in the world and has a life expectancy of 48 years at birth (UNDP, 2011). Although Sierra Leone was a British colony, English remains the primary written language taught in schools and Krio is the *lingua franca* in many healthcare settings. Kono District is a region located about 7 hours east of Freetown near the Guinea border, and the centre of the Sierra Leone diamond mining industry. *Kono* is also an indigenous language and is the term used to describe the Kono-speaking inhabitants of the region. About 100,000 of 400,000 people in Kono live in the district capital, Koidu City.

Sierra Leone has an HIV prevalence of 1.5% (UNAIDS, 2015b). The national ART programme was established in 2005 and has provided free HIV medications to all HIV-infected persons. During implementation of our programme, the national guidelines recommended that ART be initiated in the following clinical situations: (1) WHO clinical stage 3 or 4 or (2) WHO clinical stage 1 or 2 with a CD4 cell count less than 350 cells/mL. Kono District has eleven ART clinics, but surveillance data from the National AIDS Control Program reported that Koidu Government Hospital and the Dorma Clinic, also in Koidu City, deliver ART to over 85% of the population who needs ART across the district. Our programme included patients from these two ART clinics. In addition to the standard of care, we implemented community-based ART as a pilot programme in Sierra Leone.

### Description of standard of care

Aside from the provision of free ART, HIV care is also free. Persons newly diagnosed with HIV infection are staged for ART at the first clinical visit. Those HIV-infected persons in need of ART can be initiated with a plan for ART counselling at the next available appointment. Because ART clinics in Kono District have a relatively low patient volume, there is greater co-localisation of services, and ART counselling can occur in the same visit as ART initiation. Regardless of need for ART, all HIV-infected persons are provided prophylaxis for opportunistic infections (i.e. cotrimoxazole) and are expected to attend monthly visits where medication refills and routine clinical monitoring occurs.

### Description of community-based ART programme

**Figure 1.**
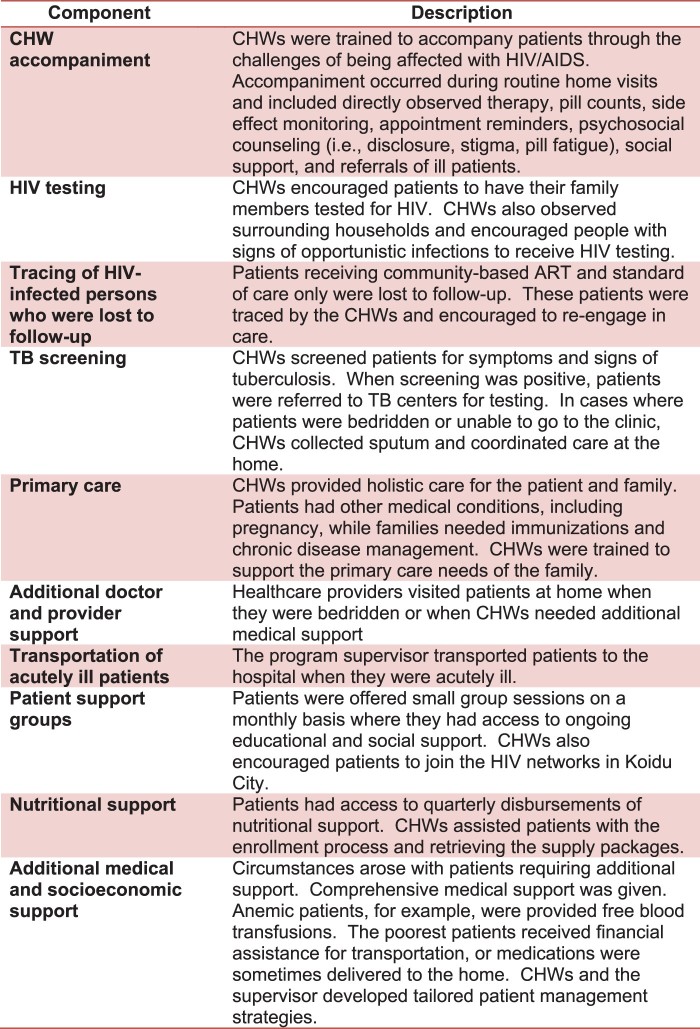
Components of the community-based ART programme and their descriptions.

In 2009, HIV/AIDS was highly stigmatised in Koidu City, and ethnographic research carried out by organisational collaborators revealed many stories of patients who faced extreme marginalisation, ostracisation and isolation after being diagnosed – several even died at home. This research prompted us to develop a community-based ART pilot programme that would strengthen the standard of care through a comprehensive, patient-centred, case management strategy. This strategy included medical, educational, psychological, social, and economic support (Figure 1). A community health worker (CHW) and supervisor worked collaboratively to develop the management strategy.

Criteria for CHW selection were as follows: HIV-infected; a member of the community; and fluent in *Kono* and *Krio* as well as able to read and write English (since training materials and monitoring tools were only able to be produced in English). Final selection was based on results of the training. The training was conducted with the Partners in Health *Accompagnateurs* Training Guide (2007). The manual was tailored to the sociocultural context within Sierra Leone. The training was 10 days in length and was followed by an intensive field training module.

CHWs were given reporting forms to monitor adherence and appointments of their patients, and to facilitate other patient case management issues. Field supervision ranged from a weekly to monthly observation. Unscheduled supervisory visits were conducted to ensure patient satisfaction and quality service delivery. Each CHW was compensated USD 50 per month for their work.

In the planning phases of our pilot programme, we needed to develop an initial strategy for enrollment because we did not have sufficient funding to offer the HIV programme to the entire HIV community in Koidu City. Therefore, we opted to randomly select HIV-infected persons on ART who were living in Koidu City and then invite them to join our programme. We obtained a list of names from the National AIDS Control Program and two local HIV support groups. Both ART-naïve and ART-experienced persons were included. Prior to selecting names, we excluded persons who were not intending to continue their routine care in Koidu City, were living outside the geographic coverage area of the CHWs, or became a community health worker. The remaining names were placed in a hat, and local HIV support group witnessed the names as they were drawn from the hat. Selected names were invited to join our pilot programme. Subsequent to this initial selection process, the CHW supervisor created a waitlist in collaboration with the ART clinic staff. All HIV clinic patients were eligible to join the programme, and the waitlist was sequentially developed as patients presented to clinic. Each name was associated with a number. Over time, our programme expanded and we had attrition of participants. When we were able to invite more HIV-infected persons to join our pilot programme, we used the waitlist to invite additional potential participants. Patients were enrolled based on the numerical order of the waitlist.

From November 2009 to September 2011, CHWs were instructed to perform directly observe therapy (DOT) during home visits and occur twice daily for seven days every week. In October 2011, home visits were reduced; they continued to occur once daily three times every week. A typical CHW work schedule was to visit participants on Mondays, Wednesdays, and Fridays. At the first visit of the week, CHWs counted pills to assess adherence to ART. During the other two visits per week, the programme placed emphasis on providing non-medical support (Figure 1). Other changes made to the programme after September 2011 included reducing the geographic coverage area of the CHWs, enrolling new participants, increasing the number of participants from 10 to between 20 and 30 per CHW, and training and hiring of more CHWs. We also worked with CHW supervisors to strengthen their role managing CHWs in order to optimise the health and wellbeing of participants. A programmatic culture of collaborative problem solving, sharing success stories as well as challenges, and social justice advocacy was cultivated amongst the teams of CHW supervisors and CHWs. A timeline of the interventions can be found in Table 1.

The programme considered ‘real-world’ circumstances and allowed for flexibility in scheduling and activities of home visits based on the needs and interests of the participants. For example, the patient-centred approach to case management allowed for more social support if participants did not have any retention or adherence issues. Other situations arose such illness that required unscheduled CHW attention. By strengthening supervisory systems for CHWs and making the abovementioned and other changes to the programme, we intended to balance flexibility, independence, and structure with the goal that we would continue to achieve the same level of comprehensive care as the more intensive version of the programme.

### Data collection

Our primary outcome was retention in care, defined as having attended a clinic visit in the last 150 days (Chi et al., 2011). Adherence to ART was a secondary outcome and measured using the following two methods in the study: (1) by directly observed therapy, which was further categorised into observed and unobserved doses and (2) by pill counts using the following calculation: 1 – (number pills remaining between counts/number pills prescribed between counts).

While we measured adherence to ART for our programme participants throughout the study period, we only measured adherence to ART for a sub-set of individuals receiving standard of care during a three-month period. Additional details of this study are published elsewhere and focus on the adherence to ART for those individuals who were only receiving standard of care (Kelly et al., 2013). In this study, we focused on adherence measurement with unannounced pill counts for our study groups (standard of care and community-based ART) over a three-month period (Table 1).

Facility-based data were collected from the ART registers at Koidu Government Hospital and Wellbody Clinic. Community-based data were collected from the reporting forms of the community health workers. Data from the community-based ART and standard of care groups were time-matched to allow for comparison. Facility-based data were censored at 24 months.

### Data analysis

Age, sex, and ART initiation were demographic data available from the ART registries. We used these variables to test for differences between groups in and out of care as well as between groups receiving standard of care with and without community-based ART. Fisher’s exact tests were conducted to determine if there were significant differences in retention outcomes at 12 and 24 months. Three months of adherence data were calculated for persons who received community-based ART (at different home visit frequencies) and for persons who received standard of care. Adherence was analysed as a continuous variable, and adherence determinations of >100% were truncated at 100. Mean and median adherence of the study population was calculated. Student *t*-tests were used for analysis. Data was analysed in STATA/IC 14.1 (STATA Corporation, College Station, TX, USA).

### Ethical approval

We initiated our pilot programme for patient care, not for human subjects research. We obtained ethical approval by the Sierra Leone Ethics and Scientific Review Committee and the Institutional Review Board at Baylor College of Medicine to use programmatic and clinic records for research purposes.

## Results

Between November 2009 and February 2011, 52 persons were enrolled in the programme and were initiated on community-based ART. During the same time frame, 91 persons who were ART-naive or ART-experienced were enrolled and only received standard of care. No significant differences in age, gender, or number of ART-experienced persons were observed among groups who were retained in care and LTFU as well as among groups who received standard of care with and without community-based ART.

### Retention in care at 12 months

Of the 52 HIV-infected persons who were receiving community-based ART with home visits twice daily, 32 persons (61.5%) were retained in care at 12 months. During the same time period, 29 (31.9%) of 91 persons only receiving standard of care were retained in care. Participants receiving standard of care plus community-based ART were significantly more likely to be retained in care (*p* < .01) than those who were only receiving standard of care. Comparative outcomes at 12 months are presented in Table 2.

**Table 2.** Retention rates at 12 months and 24 months of HIV-infected persons receiving standard of care compared with those receiving the addition of community-based ART.

	Community-based ART (*n* = 52, %)	Standard of care (*n*= 91, %)	*p*-Value
Retained in care at 12 months	32 (61.5%)	29 (31.9%)	<.01
Re-engaged in care	6	12	
Retained in care at 24 months	38 (73.1%)	41 (44.0%)	<.01

### Retention in care at 24 months

Of the 32 persons retained in care from the community-based ART programme, one person became a community health worker and was excluded from further analysis. Thirty-one persons had their home visits transitioned from twice daily every day per week to once daily three times every week and were observed for another 12-month period. After 12 additional months, all 31 persons (100%) were retained in care, and 6 persons who had been lost to follow-up re-engaged in care. The 24-month retention rate (from initiation of community-based ART to 12-month censor after the taper) was 73.1% for participants in the community-based ART programme. In the standard of care group, 29 persons continued in care when the community-based ART programme was transitioned from twice daily to three times per week. After observing this group for another 12-month period, 28 persons (96.6%) were retained in care, and 12 persons who had been lost to follow-up re-engaged in care. The 24-month retention rate was 44.0% for participants in the standard of care group. At 24 months, the community-based ART programme continued to demonstrate significantly higher retention rates than the standard of care only (*p* < .01) (Table 2).

### Adherence to ART over a three-month period

Thirty-two persons who received community-based ART with home visits twice daily had a mean and median observed adherence (SD) of 89.4% (6.9) and 91.1%, respectively. Thirty-one (96.9%) of these persons who received community-based ART with home visits three times per week had a mean and median adherence (SD) of 85.5% (8.4) and 86.8%, respectively. In the standard of care group, 58 persons had a mean and median adherence (SD) of 78.6% (18.2) and 81.8%, respectively. Mean observed adherence for persons who received community-based ART with home visits twice daily every day per week was significantly higher than persons receiving the standard of care only (*p* = .02). Mean adherence for participants of the community-based ART programme who received home visits once daily three times per week was also significantly higher than mean adherence for participants who only received standard of care (*p* = .03). Differences in mean adherence among variations of community-based ART and the standard of care are presented in Table 3.

**Table 3.** Differences in mean adherence comparing standard of care against community-based ART (*p-values).

	Standard of care (*n* = 58)	Home visits twice daily every day per week (*n* = 32)	Home visits once daily three times per week (*n* = 31)
Standard of care	1	.02*	0.03
Home visits twice daily every day per week	** **	1	0.08
Home visits once daily three times per week	** **	** **	1

## Discussion

We found that our community-based ART pilot programme may have improved retention and adherence outcomes when added to the standard of care in Koidu City, Sierra Leone. Although some community-based studies have shown less potent findings (Gross et al., 2009; Wohl et al., 2006), the impact of community-based ART is somewhat specific to the individual and population (Farmer et al., 2001; Hart et al., 2010). Our pilot programme adds to a growing body of evidence, suggesting that longer-term community-based ART may contribute to the positive HIV health outcomes needed to reach the UNAIDS goal of 90-90-90.

In Kono District, the retention and adherence rates from those receiving standard of care were lower than global means (Fox & Rosen, 2010; Mills et al., 2006), and a national assessment (Dalan Development Consultants, 2011). Our findings in Koidu City may have been different than global and national estimates for various reasons, including a weak health system, nascent government HIV programme, high levels of HIV stigma, and other structural and social barriers to HIV care (Cancedda et al., 2016; Kelly, Reid, Lahiff, Tsai, & Weiser, 2017; Kelly et al., 2016). Our programme was designed to respond comprehensively to many of these issues. CHWs accompanied participants to their clinical appointments and drove them the clinic when they were sick. CHWs also addressed stigma, provided social and food support, counselled patients on substance abuse, recognised issues of medication toxicities, and educated communities and participants on HIV/AIDS and the value of care (Figure 1). They cultivated bonds of solidarity with patients and these enduring relationships helped address the isolation many HIV patients face after abruptly receiving a diagnosis. Other community-based ART programmes have the potential to address any number of these barriers, even for periods as long as 5 years, depending on the extent to which they exist in different settings around the world (Fatti et al., 2012). While our community-based ART programme addressed many of the barriers observed, the programme could be expanded to address other barriers with interventions focusing on substance abuse counselling, transportation benefits, additional nutritional support, employment opportunities, and enhanced reminder systems.

Adherence outcomes were positively correlated with community-based ART regardless of the frequency of home visits, and these findings may guide the type and frequency of CHW support provided through community-based ART programmes. Someone with substance dependence to alcohol could benefit from home visits twice daily while someone with sustained excellent adherence and no known barriers to HIV care might not need home visits three times per week. Persons with substance dependence, for example, who require more frequent visits could benefit from co-delivered interventions and, if successful, these interventions could change behaviors and reduce the need for more frequent visitations. Studies have suggested that sustaining the benefit of this programme may require continuation of home visits (Hart et al., 2010), but additional work needs to be done to understand the balance of home visit frequency and activities.

This study has limitations. When initiating and rolling out this pilot programme, we faced funding constraints and ethical issues that limited our sample size but created a quasi-experimental study design within the HIV-infected population living in Koidu City. Therefore, the findings of our study should be interpreted with caution. Our study had selection biases. We included ART-experienced persons and used a waitlist as our selection procedure for additional participants to join the programme over time. It was logistically challenging to initiate community-based ART at the same time as the initiation of ART. Initiation of community-based ART often lagged, but retention outcomes were long term and adherence measurements were obtained at least 9 months after the community-based ART programme had started. Sierra Leone did not have access to viral load facilities during the study period. However, most of these threats to validity would bias towards the null and the findings were statistically significant. Although we attempted to time-match groups in our retrospective, quasi-experimental study design, stronger study designs from the field of implementation science (i.e. step-wedge approaches) are needed to assess the effect of tapering community-based ART over time in settings similar to ours in Sierra Leone.

In summary, a pilot programme in Koidu City provides new evidence that community-based ART has the potential to improve retention and adherence outcomes for a group of HIV-infected persons, regardless of the frequency of home visits. Overcoming the barriers to HIV care requires a comprehensive, patient-centred approach that may include clinic-based and community-based strategies. In rural Sierra Leone, HIV patients routinely face a myriad of retention and adherence challenges, suggesting that a comprehensive but flexible patient-centred approach to community-based ART may be a viable intervention. Using these findings, the National HIV/AIDS Secretariat in Sierra Leone opted to use our community-based ART pilot programme as the model for national scale-up of community-based HIV care.
